# A healthy diet with and without cereal grains and dairy products in patients with type 2 diabetes: study protocol for a random-order cross-over pilot study - Alimentation and Diabetes in Lanzarote -ADILAN

**DOI:** 10.1186/1745-6215-15-2

**Published:** 2014-01-02

**Authors:** Maelán Fontes-Villalba, Tommy Jönsson, Yvonne Granfeldt, Lynda A Frassetto, Jan Sundquist, Kristina Sundquist, Pedro Carrera-Bastos, María Fika-Hernándo, Óscar Picazo, Staffan Lindeberg

**Affiliations:** 1 Department of Clinical Sciences, Center for Primary Health Care Research, Lund University/Region Skåne, Malmö, Sweden; 2 Department of Food Technology, Engineering and Nutrition, Lund University, Lund, Sweden; 3 Department of Medicine, Division of Nephrology, University of California San Francisco, San Francisco, CA, USA; 4 Faculty of Health Sciences, Universidad de Las Palmas de Gran Canaria, Las Palmas de Gran Canaria, Spain; 5 NutriScience-Education and Consulting, Lda, Lisbon, Portugal; 6 Calle José Betancort, 15, 35530, Teguise-Lanzarote, Spain

**Keywords:** Protocol, Random-order cross-over trial, Type 2 diabetes mellitus, Metabolic diseases, Dietary intervention, Grain-free diet, Dairy-free diet, Glucagon, Fructosamine

## Abstract

**Background:**

Research on the role of nutrition in type 2 diabetes has largely focused on macro/micronutrient composition and dietary fiber intake, while fewer studies have tested the effects of differing food choice. Some observational studies and short-term intervention studies suggest that a food pattern mimicking the diet with which humans evolved positively influences glucose control and associated endocrine systems. Such a food pattern mainly differs from other common healthy food patterns in its absence of cereal grains and dairy products. The primary aim of this pilot study is to determine the effect of two healthy diets with or without cereal grains and dairy products on glucose control, while keeping participants’ weight stable and other food parameters, such as macro/micronutrient composition, dietary fiber and glycemic load, the same in both diets.

**Methods/Design:**

We intend to include 15 adult patients with a medical diagnosis of type 2 diabetes mellitus with or without medication and with an increased waist circumference (≥ 80 cm for women and ≥ 94 cm for men) in a random-order cross-over diet intervention study during two periods of four-weeks separated by a six-week washout period. Patients will be instructed to eat two healthy diets according to official dietary guidelines with respect to macro/micronutrient composition and fiber content, but differing in the type of food included, with one diet being without cereal grains and dairy products. Lunch will be served in a hospital kitchen for control of nutrient intake, while the rest of the meals will be eaten at home according to specific directions. The energy content of the diets will be individually adjusted to maintain a stable body weight during the two four-week intervention periods. Primary outcomes will be change in fasting plasma glucagon and fructosamine, while secondary outcomes include change in fasting glucose and glycated hemoglobin, glucose and glucagon response during oral glucose tolerance test, blood lipids, blood pressure, C-reactive protein, body composition, quality of life, subjective experience with the two diets, satiety scores and changes in medication.

**Discussion:**

Using these results, we will assess the need to conduct larger and longer studies with similar design.

**Trial registration:**

This trial was registered at clinicaltrials.gov as NCT01891955 and Spanish Agency of Medication and Sanitary Products (AEMPS) registration code: MFV-ADI-2013-01.

## Background

The prevalence of type 2 diabetes mellitus (T2DM) has increased dramatically (more than doubled) since 1980, with a global prevalence of 9.8% and 9.25% for men and women, respectively, and affecting 347 million adults in 2008 [1]. In Europe, the prevalence of T2DM is estimated to be 8.1% of the adult population, affecting 52.8 million people in 2011. The estimated prevalence of T2DM for 2030 is 9.5% of the adult population affecting 64.2 million people. T2DM accounts for 85 to 90% of all cases of diabetes mellitus.

T2DM is one of the world’s most important causes of mortality, disability and economic cost due to the associated increase in the risk of micro- [2] and macrovascular [3] complications. T2DM caused 282,400 and 317,000 deaths in 2011, for men and women, respectively [4]. Estimates indicate that at least USD 131 million were spent in Europe due to T2DM in 2011 [4]. In 2003, the prevalence of T2DM in the archipelago of Canary Islands (Spain) was estimated to be 12% of the population with an economic cost of €38.8 million, which accounts for 2.1% of all the health costs of the Canary Islands’ Government [5,6]. Furthermore, micro- and macrovascular complications, including end-stage renal disease, are four to five times higher than in the rest of Spain [6,7]. In impaired glucose tolerance (IGT), lifestyle intervention reduces the incidence of T2DM [8], even when compared with insulin sensitizing medication [9]. In a study from primary health care in Stockholm, more cardiovascular risk factors were improved after an exercise intervention in people with normal glucose tolerance compared with those with IGT or T2DM [10]. However, in these studies the separate effect of exercise versus diet could not be elucidated [11]. Data from systematic reviews regarding the role of food in the prevention and treatment of T2DM indicates that there are some uncertainties with respect to the optimal dietary intervention [11,12], and results from one large interventional dietary study suggest that targeting macronutrient composition by lowering total fat intake and increasing carbohydrate intake from whole grains, may have detrimental effects in women with pre-existing T2DM [13]. A recent intensive diet and exercise program in patients with T2DM was stopped because the intervention did not reduce cardiovascular risk, despite significant weight loss at four years, compared to the control group [14]. Research on the role of nutrition in T2DM has largely focused on macro/micronutrient composition and fiber intake, while fewer studies have tested the more direct endocrine effects of food [13,15,16]. A recent systematic review and meta-analysis suggests that low-carbohydrate, low-glycemic index, Mediterranean and high-protein diets may be effective in improving cardiovascular disease risk and diabetes management compared to control diets, with no significant difference effect between the different interventions [17]. Also, the effect of weight loss over macronutrient composition precludes drawing a definitive conclusion derived from this meta-analysis.

Theoretically, dietary interventions can improve glucagon [18] and leptin physiology [19]. Thus, we speculate that the possible effects of the diets in the ADILAN study could be mediated, in part, by improving the action of those hormones.

Some observational studies [20] and two short-term intervention trials from our group [21,22] suggest that a food pattern mimicking the diet with which humans evolved, may be an optimal approach for patients with T2DM. Importantly, the short-term trials compared the experimental approach to the recommended dietary treatment in each case, namely the Mediterranean diet for patients with ischemic heart disease [21] and the American Diabetes Association diet for patients with T2DM [22], respectively. In both trials, the experimental approach resulted in better outcomes than in the control diet. A noteworthy finding in those dietary trials is the significant difference in reported macronutrient composition between interventions and controls, specifically, higher energy percentage intake for protein and fat (except in Lindeberg *et al*. [21] for fat), and lower energy percentage intake for carbohydrate in the experimental versus the control diet, taking into account that the intervention diet was not fixed to any dietary macronutrient ratio. Dietary glycemic load (GL) was also significantly lower.

Notwithstanding these findings, the beneficial effects shown in the experimental diets in both trials are not fully explained by macronutrient composition or GL after further statistical analysis [21,22]. We concluded that food choice, rather than micro-and macronutrient composition, may have been the most important factor leading to the beneficial effects observed. In a recent meta-analysis comparing Mediterranean diets to alternative dietary strategies, the authors stated that the paleolithic diet (a healthy diet without grains and dairy) in the study by Lindeberg *et al*. [21] demonstrated the most positive effect on fasting blood glucose of all the studies included in the meta-analysis [23].

### The Alimentation and Diabetes in Lanzarote (ADILAN) study

The major aim of the ADILAN study is to test the direct endocrine effect of food items beyond macro/micronutrient composition, fiber content and glycemic load of the diet. For this purpose, we will try to avoid weight loss in an attempt to isolate the effect of food, as weight loss could have been a confounding factor in the previous studies. Our objective is to run a pilot intervention for a later long-term study with a representative sample in patients with T2DM. The working hypothesis is that food choice rather than macro/micronutrient composition, fiber intake, or GL, is a major determinant for the prevention and treatment of T2DM. Hypothetically, the foods that composed the diet during most of the time of *Homo sapiens*’ evolution, may be the optimal dietary approach for the prevention and treatment of T2DM [24,25].

## Methods/Design

### Participants

We aim to enroll 15 participants with T2DM, males and females, > 18 years old based, on the power calculations (see below) estimation of a required minimum of 13 patients, allowing for one or two drop-outs. Patients live in the island of Lanzarote, in the archipelago of Canary Islands (Spain), located off the western coast of Africa.

### Recruitment

Patients will be recruited through different strategies. We are in collaboration with the *Asociación de Diabéticos de Lanzarote* (ADILA), a local organization, whose aim is helping patients to deal with the consequences of type 1 diabetes and T2DM. Data from 1996 estimated the prevalence of T2DM in Lanzarote to be 6.6% of the population aged between 15 and 75 years old, with an absolute number of 4,067 patients [26,27]. ADILA will contact the potential participants registered in their database by telephone, Email and advertisement in the association’s bulletin board. In addition, we will advertise the study in the local newspapers, radio and in some institutional websites on the island of Lanzarote. Patients interested in participating in the study will receive general oral and/or written information about the study. They will be informed that our objective is to compare two healthy diets because we do not know which, if any, is the better one. If a person is interested, we will register their name and telephone number for further contact. After recruiting 15 potential participants, a meeting will be arranged to provide further details about the study.

### Eligibility

We will include adults (> 18 years old), males and females, with a medical diagnosis of T2DM and increased waist circumference (≥ 80 cm for women and ≥ 94 cm for men), with or without medication (including insulin treatment), with stable weight for three months prior to the start of study, who have received no change in dose of beta blocker or thyroxine for three months prior to the start of study, and no anticoagulant or oral steroid treatment. Inclusion criteria are present glycated hemoglobin (A1c) ≥ 6.0% (≥ 42.1 mmol/mol), with no upper boundary, creatinine < 130 μmol/L and liver enzymes less than four times above the upper reference value.

### Procedure

**Figure 1:**
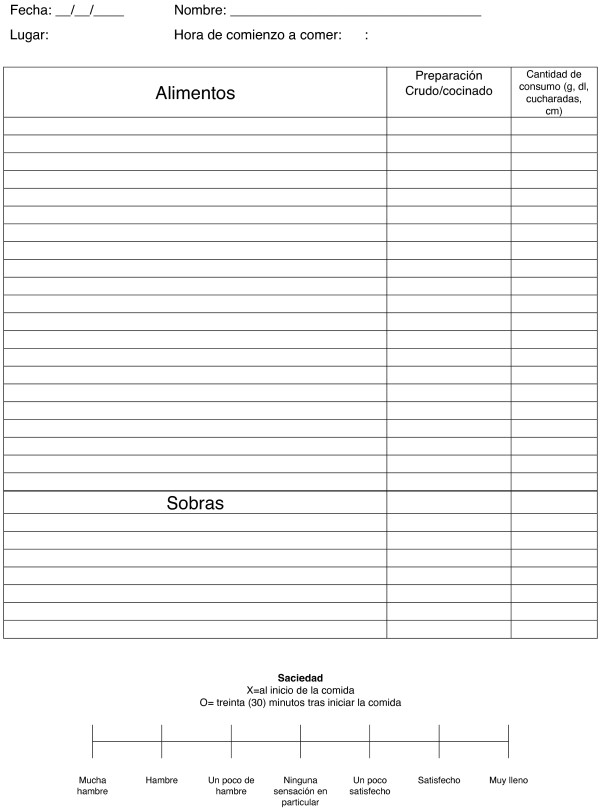
Four-day weighed food record and satiety score sheet.

The first 15 enrolled participants will undergo baseline tests, including A1c, creatinine and liver enzymes. At this time they will be instructed, in a group session, how to perform the four-day weighed food record and fill the satiety score sheets (Figure 1). Later, participants who meet the inclusion criteria will be notified about their final inclusion in the trial and reminded how to perform the four-day weighed food record and satiety score. Participants will borrow an electronic weighing scale with tare function and will be provided with enough sheets for the four-day weighed food record and satiety score. Five to seven days later, they will return the food records. The baseline four-day food record will yield information about the pre-study food choices and nutritional composition. The four-day food record and satiety score will be carried out at the start and end of each intervention period. After receiving the first food records, participants will be appointed for the first laboratory tests and intervention allocation. Based on total energy expenditure, as estimated by a bio-electrical impedance analyzer model BC-545 (Tanita Corporation, Tokyo, Japan), we will provide each participant with a menu plan with the approximate energy intake to avoid changes in body weight.

Regarding insulin titration, the participant’s physician will handle insulin titration as needed.

### Randomization

The study participants will be randomly allocated to start with either diet A or diet B. The process will be performed after the first laboratory testing. Randomization will be performed by use of an Internet-based random sequence generator from the School of Computer Science and Statistics, Trinity College, Dublin [http://www.random.org/sequences/]. Due to the small sample size, and to avoid unbalanced allocation of the participants to one of the starting diets, stratified sampling by use of minimization technique will be used. Weighting variables are starting diet and duration of T2DM [28].

## Consent

Once 15 interested persons contact the researchers, an initial meeting with the study investigators will be arranged to give additional information about the study. Additional eligible subjects will be placed in queue for possible participation in case of drop-out(s). People who are willing to participate in the study and who meet inclusion criteria (medical diagnosis of T2DM, increased waist circumference, stable body weight for three months prior to the start of study, stable dosing of beta blockers or thyroxine for three months prior to the start of study, and absence of chronic oral steroid treatment, and anticoagulant treatment) will receive written information about the study and informed consent as approved by the ethics committee. After returning the informed consent, laboratory tests for plasma A1c, creatinine and liver enzymes will be analyzed. For each person who is excluded or declines participation, one person from the queue will be contacted and informed individually.

### Ethics committee and participants insurance

The ADILAN study was approved by the Ethics Committee of Clinical Investigation (CEIC) of the Hospital Universitario de Gran Canaria, Doctor Negrín (Code CEIC Negrín: 130030). The study participants will be protected against any medical complication derived from the intervention, with an insurance exclusively contracted for ADILAN (HDI Seguros, policy number 130/002/001897), registered at clinicaltrials.gov as NCT01891955.

### Design

**Figure 2:**
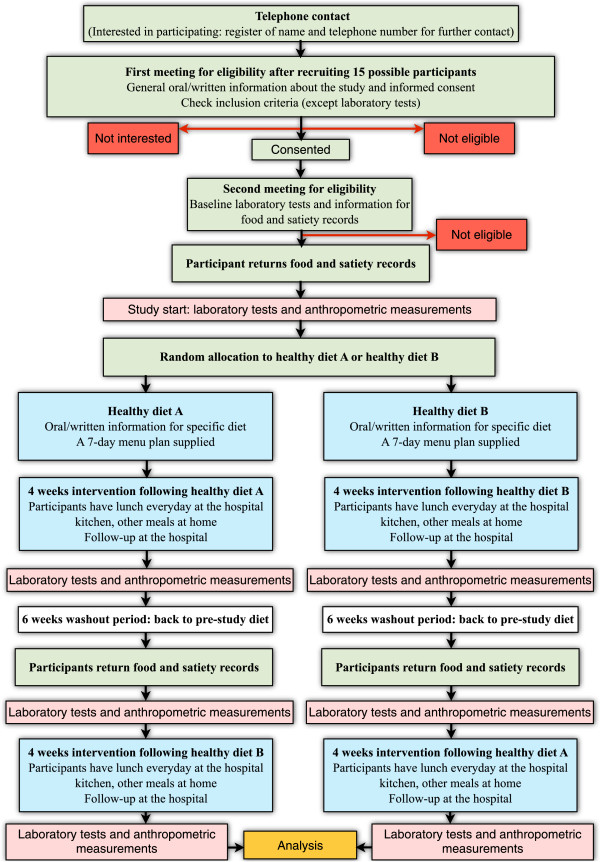
Diagram flow for Alimentation and Diabetes in Lanzarote (ADILAN).

The ADILAN study is a randomized cross-over open label dietary intervention trial in patients with T2DM who will be instructed to eat two different dietary patterns during two four-week periods separated by a six-week washout period. The procedure for screening, eligibility and recruitment is detailed in Figure 2. Schedule of enrollment, interventions and assessments can be found in Additional file 1. Detailed information about enrollment, interventions and assessments is described in Additional file 2.

### Power calculations

The predefined primary outcomes are changes in fasting fructosamine and glucagon. In order to detect, with 80% power and at a significance level of 5%, a 26 μmol/L larger reduction in fructosamine after diet B than after diet A, 12 subjects are estimated to be required. For a 37 ng/L difference in change in fasting glucagon between the diets, 13 subjects are estimated to be required.

### Intervention

The intervention is based on two diets: a) healthy diet A (healthy diet with cereal grains and dairy products), and b) healthy diet B (healthy diet without cereal grains and dairy products). After randomization, there will be no blinding to dietary assignment for study participants, nor for those administering the interventions or assessing the outcomes. Following randomization, all subjects will receive oral and written information about their respective initial dietary intervention. Owing to the fact that all participants in both groups will start the intervention at the same time, information about the specific intervention will be provided in group sessions, one for each group before the study starts. Prior to the beginning of the second dietary period, participants will be instructed about the new dietary intervention, in group sessions. Each session will last one hour and will be performed in the meeting room at the Hospital Insular de Lanzarote after the laboratory tests. The degree of behavioral support will be the same in both interventions in order to minimize differences in motivation. Written information with dietary advice, a seven-day menu plan and food recipes are similarly formulated for both diets. The rationale behind the higher intake of whole grains, dairy and legumes in healthy diet A is based on the possible beneficial effect of those foods in the treatment of T2DM in some studies [29], and their prominent position in Spanish dietary guidelines for patients with T2DM [30]. The rationale behind the exclusion of grains, dairy and legumes in healthy diet B is based on the observed positive effects in some previous studies [21,22].

Written information consists of a detailed seven-day meal plan with five meals per day and corresponding recipes (Additional file 3). Participants will be requested to follow the menu plan as much as possible when eating away from home. They will be requested to report (in as much detail as possible, the ingredients) any meal taken which differs from what is stipulated in the menu plan. In that case, they will be encouraged to photograph the meal, and send it to one of the investigators, whenever possible.

Advice about regular physical activity will be given equally to all subjects. Specifically, they will be recommended not to change their current physical activity during the whole trial.

One of the objectives of the study is to avoid weight loss during the intervention. In order to accomplish this objective, participants will be weighed once a week in order to check for weight stability. If any participant loses ≥ 1 kg compared to baseline, a new meeting will be arranged for that person to adjust his/her meal plan. We have created different versions for both menu plans in order to provide different amounts of energy per day; specifically 1,500, 1,800, 2,000, 2,200, 2,500 and 2,800 kcal/day. All the different menu versions maintain the same macronutrient composition. Depending on body weight as measured every week, each participant will be reassigned to another (with higher or lower energy) menu plan if their body weight changes by ≥ 1 kg. The meals will be taken to the lunch room in two trolleys marked with menu A or B, respectively. Each trolley will contain the trays labeled with the participant’s name. Finally, the lunch room will be separated in two areas for menu A and B, respectively.

#### Improving patients adherence to the meal plan

Despite the notion that a healthy diet can improve glucose control, adherence to dietary advice is generally poor among patients with T2DM [31]. Moreover, the particular dietary design of this trial could increase the risk of nonadherence. In order to minimize this risk, several strategies will be adopted. To facilitate the participants eating the same approximate amount of equivalent food in every meal (expressed in grams in Additional file 3), we will supply a table of equivalence. For example, 56 grams of tinned tuna equates to a small tin of tuna and 20 grams of bread corresponds to a slice of bread. Similarly, food items such as eggs or fruits will be listed as small, medium or large. In the case of more elaborated dishes, such as stewed meat, baked tubers, soups, or creams, we will provide the participants with detailed recipe directions corresponding to four servings, so as to facilitate the task in the family context. On the first day of each intervention, the investigators will review all the assigned meals, together with the participants and their partners, to make sure that the directions are understandable and easy to follow. Previous to starting the study, subjects not included in the trial will test the meals in order to elaborate a meal plan that is understandable and easy. We will ask the participants to call one of the investigators if they have any questions regarding the meal plan. Besides this, one of the investigators will meet the participants in the lunch room at the beginning of each week to give behavioral support. Furthermore, an assistant will be present on a daily basis in the lunch room to ensure that the meals are served as planned. Also, the assistant will be asked everyday to inquire whether the participants have any difficulties following the meal plans.

#### Healthy diet A

Healthy diet A will include fruit, vegetables, fish, shellfish, lean meat, nuts, eggs, olive oil and substantial amounts of whole grains, low-fat dairy products and legumes. Macronutrient composition and fiber intake will not differ from healthy diet B. GL will also be matched to that of healthy diet B. Healthy diet A is termed ‘healthy diet with cereal grains and dairy products’. This diet is classified as ‘very healthy’ using a Spanish validated nutritional software package [32]. It is in accordance with official Spanish dietary recommendations for people with T2DM regarding macronutrient composition, dietary fiber, minerals and vitamins (Tables 1 and 2). A detailed description of each of the meals proposed during the course of one week is included in Additional file 3.

**Table 1:** Dietary composition of the two healthy diets

	**Energy content, kcal (SD)**		***P-*** **value**^**a**^
	**Healthy diet A**	**Healthy diet B**	
Cereals	711.4 (105.2)	0	0.001
Legumes	185.4 (185.1)	0	0.07
Vegetables	102.6 (60.2)	421.4 (130.5)	< 0.0001
Fruits	259.4 (56.1)	980.1 (183.1)	0.001
Dairy products	216.6 (53.9)	0	0.001
Meats	62.9 (62.4)	173.1 (86.6)	0.02
Fish	165.7 (90.3)	186.4 (48.5)	0.6
Eggs	49.3 (41.7)	33.2 (41.4)	0.2
Sugars and cakes	0.97 (2.6)	11.8 (28.2)	0.6
Oils and fats	245.7 (45.3)	167.1 (43.7)	0.006
Drinks	8.9 (11.5)	11.5 (6.5)	0.6
Pre-cooked food	2.2 (3.0)	3.8 (5.1)	0.6
Snacks	7.1 (18.9)	7.0 (11.5)	0.5
Sauces and dressings	12.6 (26.7)	5.6 (4.4)	0.94
Other	0.5 (1.2)	0.5 (1.2)	1.0
Total energy	2,031.3 (142.3)	2,001.7 (59.5)	0.6
Total weight	1,866.8 (300.1)	2,099.8 (184.0)	0.1

Dietary composition (kcal) of the two healthy diets, as calculated from seven-day menu plans. Total weight in grams/day.

Values are means (SD).

^a^Mann-Whitney *U*-test, except for meat, fish, oils and fats, and sauces and dressings, total energy and total weight (*t*-test).

**Table 2:** Daily intake of macronutrients, micronutrients, fiber, glycemic load and index, cholesterol and water, as calculated from seven-day menu plans

	**Diet**		***P-*** **value**^**a**^
	**Healthy diet A**	**Healthy diet B**	
Energy			
(kcal)	2,031 (142.3)	2,002 (59.5)	0.6
(MJ)	8.5 (0.6)	8.4 (0.2)	0.6
Protein			
g	97.2 (7.6)	93.0 (4.2)	0.2
E%	19.0 (1.8)	18.3 (1.2)	0.4
Carbohydrates			
g	245.7 (18.3)	236.7 (8.4)	0.8
E%	53.0 (3.4)	53.4 (1.5)	0.8
Fiber (g)	48.5 (10.3)	53.1 (5.7)	0.3
Glycemic load (g)	116.1 (8.0)	118.0 (11.9)	0.7
Glycemic index	47.4 (1.2)	49.8 (4.7)	0.2
Total fat (g)			
g	63.6 (12.8)	62.8 (6.9)	0.89
E%	27.6 (4.6)	27.8 (2.4)	0.95
Fatty acids			
Saturated (g)	13.3 (5.2)	10.9 (2.0)	0.3
Monounsaturated (g)	30.8 (5.7)	32.1 (4.2)	0.6
Polyunsaturated (g)	10.9 (3.0)	12.5 (2.7)	0.3
Cholesterol (mg)	257.4 (106.1)	271.4 (80.1)	0.8
Water (g)	1,395.3 (296.2)	1,708.9 (240.7)	0.05
Calcium (mg)	978.3 (123.9)	591.4 (88.6)	< 0.0001
Iron (mg)	19.9 (2.8)	18.5 (2.3)	0.3
Iodine (μg)	101.7 (27.8)	143.9 (68.1)	0.15
Magnesium (mg)	570.6 (70.7)	468.6 (49.3)	0.009
Zinc (mg)	13.6 (1.8)	9.9 (2.3)	0.006
Selenium (μg)	155.7 (23.4)	126.3 (28.8)	0.057
Sodium (mg)	1,811.9 (733.9)	1,079.3 (440.7)	0.042
Potassium (mg)	3,556.0 (305.5)	6,392.9 (575.4)	0.001
Vitamin B1 (mg)	2.2 (0.3)	1.8 (0.2)	0.004
Vitamin B2 (mg)	2.2 (0.3)	1.6 (0.2)	0.001
Vitamin B3 (mg)	45.0 (5.7)	49.0 (6.9)	0.27
Vitamin B6 (mg)	2.6 (0.6)	4.2 (0.8)	0.001
Folic acid (μg)	427.6 (133.5)	506.3 (179.3)	0.37
Vitamin B12 (μg)	6.0 (1.3)	8.6 (6.3)	0.38
Vitamin C (mg)	170.1 (46.9)	434.4 (163.5)	< 0.0001
Beta-carotene (μg)	4,233.7 (1494.5)	17,194.1 (6085.9)	< 0.0001
Vitamin A (μg)	986.3 (582.8)	3,021.1 (1027.1)	0.001
Vitamin D (μg)	5.2 (3.6)	4.8 (3.2)	0.96
Vitamin E (mg)	13.0 (5.9)	23.7 (7.4)	0.01

Values are mean (SD).

^a^*t*-test, except for carbohydrate (grams), potassium and vitamin B12 (Mann–Whitney *U*-test).

#### Healthy diet B

Healthy diet B will include fruit, vegetables, fish, shellfish, lean meat, nuts, eggs and olive oil. Macronutrient composition and fiber intake will not differ from healthy diet A. GL will also be matched to that of healthy diet A. Healthy diet B will exclude cereal grains, legumes and dairy products, which will largely be replaced by root vegetables (including potatoes), vegetables, fruit and lean meat, and slightly more fish and nuts. Healthy diet B is termed ‘healthy diet without cereal grains and dairy products’. Salt intake will be lower in healthy diet B. This diet is also classified as ‘very healthy’ using validated nutritional software package [32]. It is in accordance with official Spanish dietary recommendations for people with T2DM regarding macronutrient composition, dietary fiber, minerals and vitamins (Tables 1 and 2). A detailed description of each of the meals proposed during the course of one week is included in Additional file 3.

### Dietary design

The main goal of the study is to test the hypothesis that the type of food has an effect on glucose control, independently of macro/micronutrient composition, fiber content, GL and weight loss. Therefore, we generated two different seven-day meal patterns where macro/micronutrient composition and fiber intake are similar. GL is also similar between both seven-day meal patterns. Furthermore, we aim at creating two healthy food patterns, according to official nutritional guidelines in Canary Islands, utilizing a validated nutritional software package (DIAL) [32]. The nutritional composition of the menu plan was analyzed using another validated nutritional software (Dietist XP 3.1; Kost och Näringsdata AB, Bromma, Sweden) to check for possible errors. Minimal differences were observed in the results from both programs. The distribution of macronutrient energy of diets is 19% protein, 28% fat and 53% carbohydrate, and 18% protein, 28% fat, and 53% carbohydrate for healthy diet A and healthy diet B, respectively. The amount of fiber per 2,000 kcal is approximately 48.5 g and 53.1 g in healthy diet A and healthy diet B, respectively. The GL per 2,000 kcal is 116 and 118 in healthy diet A and healthy diet B, respectively. Regarding micronutrient intake, significant differences between the two diets can be seen in Table 2. Importantly, and because one goal of the ADILAN trial is to avoid weight loss, dried fruits were regularly included instead of fresh fruits, in healthy diet B, to match the total weight of food between the two food patterns. Two recent studies have shown that substituting cereal grains for fruits and vegetables increases satiety [19,33], and therefore it may be challenging for the participants to avoid weight loss while adhering to healthy diet B.

### Evaluation

Before and after each intervention period, an oral glucose tolerance test (OGTT) will be performed in the morning after obtaining venous blood samples in the fasting state, and measurements of blood pressure, height (only at the start of intervention 1), weight, waist and hip circumference, sagittal abdominal diameter, triceps, biceps, suprailiac and subscapular skinfolds, at the start and end of both intervention periods will also be taken. The participants will ingest 75 grams of glucose and blood samples will be drawn for glucose and glucagon at 0, 15, 30, 60, 90 and 120 minutes. Areas under the curve (AUC) for plasma glucose (AUC glucose) and glucagon (AUC glucagon) will be calculated. Of great consequence, and for safety reasons, we will assess capillary glucose, using a glucometer, before performing the OGTT. If the result is greater than or equal to 190 mg/dL (10.5 mmol/L) the OGTT will not be performed. Information about participants’ quality of life will be assessed by means of the short form quality of life questionnaire SF-36v2 (standard version), using the validated Spanish version, which will be filled by the participant during each of the OGTT procedures. During the OGTT, at the end of each intervention period, patients will also be asked to give written answers to three open-ended questions about their experience with the dietary pattern that they have been following during the previous four weeks, specifically: ‘What are your thoughts about this diet?’, ‘Describe your positive and negative experiences with this diet’ and ‘How do you think this diet has affected your health?’

During both dietary interventions, free lunch will be provided every day in the kitchen of the Hospital Insular de Lanzarote. Here, our objective is two-fold: a) to control nutrient intake as much as possible and b) to give the participants behavioral support during visits to the hospital.

### Outcome assessment

#### Primary outcomes

The primary outcomes of this study are fasting fructosamine and glucagon. These outcomes will be determined after obtaining venous blood in the fasting state at start and end of each intervention period.

#### Secondary outcomes

Fasting plasma glucose, A1c, total cholesterol, LDL cholesterol, HDL cholesterol, triglycerides, high-sensitivity C-reactive protein, blood pressure, and AUCs for glucose and glucagon during OGTT comprise secondary outcomes. Quality of life, assessed with the SF-36v2 questionnaire (standard version), satiety quotient (see below), experience with the dietary patterns and change in medication are also included as secondary outcomes.

### Anthropometric measurement and laboratory tests

Trained nurses and investigators will perform the lab tests and anthropometric measurements in the Hospital Insular de Lanzarote. All measurements and laboratory tests will follow standard certified methods [34].

Standing height without shoes will be measured with a height stadiometer. Height will be reported to the nearest 1 mm.

Weight will be measured using an electronic weighing scale placed on a hard surface with the patient in the fasting state. Patients will be asked to take off their shoes, jackets, sweaters, and any other heavy clothing, and to remove all heavy items from their pockets. Then the patients will place both feet in the center of the platform and distribute their weight evenly between both feet. Weight is reported to the nearest 0.1 kg.

Waist circumference will be measured using a measuring tape immediately above the iliac crest. Measurements will be reported to the nearest 0.1 cm.

The hip circumference will be measured using a measuring tape at the level of the trochanter major and reported to the nearest 0.1 cm.

Sagittal abdominal diameter will be measured using a sagittometer (that is, a sliding beam caliper with a ruler) in the sagittal plane during a normal expiration at the level of iliac crest (L4 to L5) with the subject in supine position on a firm bench with the knees bent. Measurements will be reported to the nearest 0.1 cm.

Blood pressure will be measured in the upper arm (preferably right arm) at the level of the heart after five minutes rest in the sitting position (MONICA criteria) [34]. Ideally, the same person should perform all measurements in the same patient, using the same sphygmomanometer and procedure. The mean of two measurements will be calculated as the final result.

Skinfold measurements will be taken by use of a validated caliper with a precision of 0.2 mm. The following points will be measured as follows:

•tricipital skinfold: in the posterior part of the arm in the middle point between the lower border of the acromion and the vertex of the olecranon.

•bicipital skinfold: in the anterior part of the arm in the middle point between the most external and superior border of the acromion and the most external and superior of the radial bone head.

•suprailiac skinfold: approximately 2.5 cm above iliac crest in mid-axillary line.

•subscapular skinfold: in the inferior vertex of the scapular bone.

### Four-day weighed food records and satiety scores

A four-day weighed food record, including one weekend day, with weighing of each food item on an electronic weighing scale (that could be set to zero), will be completed by the participants before the start and end of each intervention period (Figure 1). By completing each four-day weighed food record, participants will be asked to rate, on a scale from 0 to 100, how well the record represents their food habits in the preceding four weeks. Nutrient compositions will be assessed with the nutritional software package DIAL [32] used to create the meal plans. In parallel with the four-day weighed food records, the participants will record their subjective rating of satiety by means of a seven-point Likert-type scale (Figure 1). Satiety Quotients will be calculated, as the intra-meal quotient of change in satiety during meal and consumed energy or weight of food and drink for that specific meal.

### Data management

Study data will be collected and managed using REDCap™ (Research Electronic Data Capture) electronic data capture tools hosted at Lund University [35]. REDCap™ is a secure, web-based application designed to support data capture for research studies, providing: 1) an intuitive interface for validated data entry; 2) audit trails for tracking data manipulation and export procedures; 3) automated export procedures for seamless data downloads to common statistical packages; and 4) procedures for importing data from external sources.

REDCap™ will be configured according to the study protocol in a way that the researchers can register all data at baseline, 4, 10 and 14 weeks after study start. Only the researchers and the hospital nurses will have access to REDCap™. This web application allows for management of the trial without violation of privacy or participant confidentiality and integrity of allocation concealment. The procedure also facilitates supervision from abroad by some of the investigators.

### Data analysis

Differences in baseline characteristics between the participants will be measured using two-tailed unpaired *t*-tests. A two-tailed paired *t*-test will be used to analyze treatment effects within subjects and a two-tailed unpaired *t*-test will be used to compare mean values of outcome variables between the two diets. Normal Q-Q plots and Shapiro-Wilk test will be performed to check for normal distribution of data. Data transformation will be applied in case the data does not present normal distribution. To test for a possible period effect a two-tailed unpaired *t*-test comparing the mean difference between first and second period for outcome variables and food intake will be performed. To test a possible carry-over effect a two-tailed unpaired *t*-test comparing the average of observations for outcome variables and food intake between the two periods will be performed. *P* < 0.05 will be considered to indicate statistical significance.

Analysis will be conducted by use of SPSS for Mac Version 20 (IBM SPSS Statistics for Mac, Version 20.0. Armonk, NY: IBM Corp.)

## Discussion

As discussed previously, there is uncertainty as to what is the optimal dietary approach for the prevention and treatment of T2DM [11,12,17], and previous trials from our group raise the question of whether focusing on the type of food, rather than macro/micronutrient composition, dietary fiber and GL is a better approach [21,22]. The ADILAN study aims at comparing the effects of two healthy diets on the control of blood glucose, with no significant differences in macro/micronutrient composition, dietary fiber and GL, only different food choice. Importantly, we will try to eliminate weight loss as a confounding factor.

Of paramount importance, the nutritional software package (DIAL) used to create the seven-day menu plan generates a ‘Healthy Alimentation Index’ that classifies both diets as ‘very healthy’. Due to its specific design, constructed on evolutionary principles, the grain/dairy-free diet scored zero in two items, ‘Cereals and legumes’ and ‘Dairy’, but in spite of this, it was classified as very healthy owing to the fact that it scored the maximum (except for ‘Variety of foods with a score of 7/10) in the rest of items’, namely ‘Vegetables’, ‘Fruits’, ‘Meat, Fish and Eggs’, ‘Energy derived from fat’, ‘Energy derived from saturated fats’, ‘Cholesterol’, ‘Dietary sodium’ and ‘Variety of foods’ [32]. It can be concluded that the ADILAN study will compare two healthy diets which are similar in macro/micronutrient composition, fiber intake and GL, for the treatment of T2DM but differing in the intake of cereal grains, legumes and dairy products.

The ADILAN study could serve as a pilot study to run long-term dietary intervention trials in the future, focusing on food choice, which could shed light on a better dietary approach for prevention and treatment of T2DM.

## Trial status

The ADILAN study is currently under recruitment process at 22 October 2013.

## Abbreviations

A1c: Glycated hemoglobin; ADILA: Asociación de Diabéticos de Lanzarote; ADILAN: Alimentation and Diabetes in Lanzarote; AUC: Area under curve; GL: Glycemic load; IGT: Impaired glucose tolerance; MONICA: World Health Organization’s Multinational Monitoring of Trends and Determinants in Cardiovascular Disease protocol; OGTT: Oral glucose tolerance test; REDCap®: Research Electronic Data Capture; T2DM: Type 2 diabetes mellitus.

## Competing interests

The authors declare that they have no competing interests.

## Authors’ contributions

MFV designed the study, created the meal plans, arranged and participated in meetings with the hospital staff, worked in the web application for data capture, was responsible for obtaining funding for the study, performed the statistical analysis and wrote the manuscript. TJ participated in the study design, helped to create the meal plans, worked in the web application for data capture, helped in the statistical analysis and revised the manuscript for important intellectual content. YG participated in the study design, helped to create the meal plans, helped in the statistical analysis and revised the manuscript for important intellectual content. LAF helped to create the meal plans, participated in the study design and revised the manuscript for important intellectual content. JS revised the manuscript for important intellectual content. KS revised the manuscript for important intellectual content. OP participated in the study design, helped to create the meal plans, helped in the statistical analysis and revised the manuscript for important intellectual content. PCB revised the manuscript for important intellectual content. MFH participated in the study design, participated in meetings with the hospital staff and revised the manuscript for important intellectual content. SL participated in the study design, helped to create the meal plans, participated in meetings with the hospital staff, helped in the statistical analysis and participated in the design of the manuscript as well as revising it for important intellectual content. All authors read and approved the final manuscript.

## Supplementary Material

Additional file 1 Schedule of enrollment, interventions, and assessments. Click here for file

Additional file 2 **Detailed study diagram flow.** Detailed study diagram flow of the different events with corresponding laboratory tests and measurements at each time point. Click here for file

Additional file 3 **Seven-day menu plan.** Detailed daily description of each of the proposed meals with specific weights for both diets in a period of one week. Click here for file
