# Supplementary material for: A healthy diet with and without cereal grains and dairy products in patients with type 2 diabetes: study protocol for a random-order cross-over pilot study - Alimentation and Diabetes in Lanzarote -ADILAN
Source: Trials. 2014 Jan 2;15:2. doi: 10.1186/1745-6215-15-2 (PMC3884016; doi:10.1186/1745-6215-15-2)
Supplement: Additional file 1 — Schedule of enrollment, interventions, and assessments. [file 1745-6215-15-2-S1.docx]

**Table 1.** Schedule of enrollment, interventions, and assessments

|  | E | B1 | A | STUDY PERIOD | | | | | | | | | | | |
| --- | --- | --- | --- | --- | --- | --- | --- | --- | --- | --- | --- | --- | --- | --- | --- |
|  |  |  |  | Post-allocation intervention 1 | | | | Close-out 1 | Wash-out | B2 | Post-allocation intervention 2 | | | | Close-out 2 |
| TIMEPOINT (weeks) | -t_12_-t_2_ | -t_1_-t_0_ | 0 | t_1_ | t_2_ | t_3_ | t_4_ | t_5_ | t_5_-t_10_ | t_10_ | t_11_ | t_12_ | t_13_ | t_14_ | t_15_ |
| ENROLLMENT: |  |  |  |  |  |  |  |  |  |  |  |  |  |  |  |
| Eligibility screen | X |  |  |  |  |  |  |  |  |  |  |  |  |  |  |
| Informed consent | X |  |  |  |  |  |  |  |  |  |  |  |  |  |  |
| Inclusion lab test | X |  |  |  |  |  |  |  |  |  |  |  |  |  |  |
| Allocation |  |  | X |  |  |  |  |  |  |  |  |  |  |  |  |
| INTERVENTIONS: |  |  |  |  |  |  |  |  |  |  |  |  |  |  |  |
| Healthy diet A |  |  |  |  |  |  |  |  |  |  |  |  |  |  |  |
| Healthy diet B |  |  |  |  |  |  |  |  |  |  |  |  |  |  |  |
| ASSESSMENTS: |  |  |  |  |  |  |  |  |  |  |  |  |  |  |  |
| OGTT |  |  |  | X |  |  |  | X |  |  | X |  |  |  | X |
| Blood samples |  |  |  | X |  |  |  | X |  |  | X |  |  |  | X |
| Anthropometric measurements |  |  |  | X |  |  |  | X |  |  | X |  |  |  | X |
| Four  -day food and satiety records |  | X |  |  |  |  | X |  |  | X |  |  |  | X |  |
| SF-36 questionnaire |  |  |  | X |  |  |  | X |  |  | X |  |  |  | X |
| Open-ended questions |  |  |  |  |  |  |  | X |  |  |  |  |  |  | X |

Legend

A: Allocation.

B1: Baseline 1.

B2: Baseline 2.

E: Enrollment.

OGTT: oral glucose tolerance test.

In green, participants starting with diet A and in red participants starting with diet B.
