# Supplementary material for: A healthy diet with and without cereal grains and dairy products in patients with type 2 diabetes: study protocol for a random-order cross-over pilot study - Alimentation and Diabetes in Lanzarote -ADILAN
Source: Trials. 2014 Jan 2;15:2. doi: 10.1186/1745-6215-15-2 (PMC3884016; doi:10.1186/1745-6215-15-2)
Supplement: Additional file 3 — Seven-day menu plan. Detailed daily description of each of the proposed meals with specific weights for both diets in a period of one week. [file 1745-6215-15-2-S3.docx]

**HEALTHY DIET A**

**MONDAY**

Breakfast

- Skimmed milk (200 g)
- Gofio (45 g)
- Apple (200 g)
- Wheat bran (6 g)

AM snack

- Tinned tuna (56 g)
- Whole grain bread (80 g)

Lunch

- Leek soup (leek 144 g; carrot 150 g; garlic 1.5 g; potato 120 g; olive oil 19.5 g; black pepper 0.5 g; pinch of salt; water 250 ml)
- Baked hake with vegetables (hake 150 g; prawns 40 g; green beans 28 g; squash 25 g; onion 67.5 g; small onion 19 g and white wine 38 g)
- Whole grain bread (40 g)
- Apple (200 g)

PM snack

- Whole grain biscuit (25 g)
- Skimmed yogurt (125 g)

Dinner

- Chicken and pasta soup (chicken 150 g; water 250 g; pasta 25 g; laurel 0.2 g; onion 17 g; garlic 0.5 g and olive oil 7 g)
- Skimmed milk (200 g)
- Gofio (45 g)
- Wheat bran (6 g)

**TUESDAY**

Breakfast

- Fresh goat cheese (30 g)
- Skimmed milk (125 g)
- Whole grain bread (80 g)
- Apple (150 g)

AM snack

- Whole grain toast (60 g)
- Chicken breast-cold meat (40 g)
- Olive oil (14 g)

Lunch

- Chick pea soup (chickpea 84 g; spinach 42 g; potato 42 g; egg 10 g; white bread 3.5 g; olive oil 5 g; parsley 1g; ham 5 g; garlic 1 g and water 250 g)
- Baked chicken thighs (chicken thighs 115 g and olive oil 10 g)
- Orange (180 g)

PM snack

- Skimmed milk (200 g)
- Gofio (45 g)
- Wheat bran (6 g)
- Almonds (20 g)

Dinner

- Plate of (potato 100 g; beans 30 g; egg 60 g and tinned tuna 56 g)
- Banana (160 g)

**WEDNESDAY**

Breakfast

- Skimmed milk (200 g)
- Gofio (45 g)
- Wheat bran (6 g)
- Pear (170 g)
- Orange (180 g)

AM snack

- Whole grain bread (60 g)
- Fresh goat cheese (50 g)
- Hazelnuts (25 g)

Lunch

- Fish croquette (wholegrain wheat 17 g; skimmed milk 125 g; egg 10 g; tuna 25 g; butter 8.5 g; olive oil 22.5 g; wholegrain bread 17 g and black pepper 0.7 g)
- Salad (lettuce 50 g; tomato 75 g; onion 30 g; cucumber 50 g and vinegar 5 g)
- Peach (150 g)

PM snack

- Skimmed milk (200 g)
- Gofio (45 g)
- Wheat bran (6 g)

Dinner

- Leek soup (water 200 g; carrot 15 g; potato 15 g; pea 15 g; leek 40 g; green bean 15 g and turnip 10 g)
- Vegetable sandwich (wholegrain bread 80 g; lettuce 15 g; tomato 25 g; asparagus 20 g; tinned tuna 100 g; mayonnaise 10 g and butter 5 g)

**THURSDAY**

Breakfast

- Skimmed milk (200 g)
- Gofio (45 g)
- Wheat bran (6 g)
- Almond (20 g)

AM snack

- Tinned tuna (56 g)
- Whole grain bread (80 g)

Lunch

- Chickpea soup (chickpea 125 g; onion 67.5 g; tomato 125 g; red bell pepper 70 g; pork meat 12.5 g; potato 125 g; white wine 25 g; egg 30 g; laurel 0.5 g; water 125 g and olive oil 25 g)
- Melon (150 g)

PM snack

- Skimmed yogurt (125 g)
- Wholegrain biscuit (25 g)

Dinner

- Spaghetti (spaghetti 50 g; minced meat 30 g; onion 10 g; tomato 35 g; carrot 10 g; olive oil 5 g and water 110 g)
- Orange (125 g)

**FRIDAY**

Breakfast

- Skimmed milk (200 g)
- Gofio (45 g)
- Wheat bran (6 g)
- Apple (150 g)

AM snack

- Whole grain toast (60 g)
- Chicken breast-cold meat (40 g)

Lunch

- “Moros y cristianos” (black bean 75; rice 38 g; laurel 0.5 g; olive oil 3.5 g; parsley 1.25 g, onion 67.5 g: garlic 1.5 g and salt 0.5 g)
- Hake in green sauce (hake 250 g; water 62.5 g; parsley 12.5 g; garlic 7.8 g and olive oil 8.5 g)

PM snack

- Skimmed milk (200 g)
- Gofio (45 g)
- Wheat bran (6 g)

Dinner

- Scrambled eggs with green asparagus (green asparagus 125 g; egg 65 g; garlic 2 g and olive oil 15 g)
- Orange (180 g)
- Apple (150 g)

**SATURDAY**

Breakfast

- Skimmed milk (200 g)
- Gofio (45 g)
- Wheat bran (6 g)
- Apple (200 g)

AM snack

- Wholegrain bread (80 g)
- Tinned tuna (82 g)

Lunch

- “Arroz a la cubana”-Cuban rice (rice 35 g; tomato 30 g; water 66 g; egg 60 g; olive oil 20 g; banana 225 g)
- Wholegrain bread (40 g)

PM snack

- Skimmed yogurt (125 g)
- Wholegrain biscuit (25 g)

Dinner

- Baked “dorada”-Gilt head (gilt head 200 g; onion 25 g; olive oil 10 g; tinned skinless tomato 160 g; olives 17 g; lemon 33 g; red bell pepper 60 g; sugar 2 g; black pepper 1 g; oregano 0.5 g; garlic 1.7 g and white wine 40 g)
- Apple (200 g)

**SUNDAY**

Breakfast

- Skimmed milk (200 g)
- Gofio (45 g)
- Wheat bran (6 g)
- Orange (180 g)
- Pear (110 g)

AM snack

- Whole grain toast (60 g)
- Chicken breast-cold meat (40 g)

Lunch

- Vegetable soup with fresh cheese (chickpea 84 g; onion 16.5 g; garlic 1.7 g; egg 10 g; olive oil 17 g; spinach 25 g; green bell pepper 8.5 g; bread 14 g; parsley 0.5 g; pepper 0.5 g; water 250 g and 50 fresh goat cheese)
- Wholegrain bread (40 g)
- Pear (110 g)

PM snack

- Skimmed milk (200 g)
- Gofio (45 g)
- Wheat bran (6 g)

Dinner

- Salad (lettuce 50 g; tomato 75 g; olive oil 10 g and vinegar 5 g)
- Baked salmon (salmon 150 g; dill 1g and lemon juice 15 g)
- Wholegrain bread (80 g)
- Banana (125 g)

**HEALTHY DIET B**

**MONDAY**

Breakfast

- Avocado (40 g)
- Tomato (25 g) with oregano and olive oil (5 ml)
- Banana (125 g)
- Dried figs (25 g)
- A tin of tuna with oil (56 g)

AM snack

- Avocado (40 g)
- Banana (160 g)
- Dried plum (75 g)

Lunch

- Salad (lettuce 50 g, tomato 75 g, onion 30 g, cucumber 50 g)
- Grilled tubers (potato 125 g, sweet potato 100 g, carrot 75 g, pumpkin 75 g)
- Grilled hake (fish 150 g and olive oil 10 g)
- Boiled kale (125 g)
- Pear (170 g)

PM snack

- Strawberries (100 g)
- Almonds (15 g)
- Raisins (15 g)

Dinner

- Sweet potato pure (sweet potato 250 g, onion 75 g, pepper 75 g, olive oil 5 g, water 15 ml, black pepper 2 g)
- Grilled turkey breast (turkey breast 150 g)
- Papaya (100 g)
- Dried apricot (15 g)

**TUESDAY**

Breakfast

- Tomato (50 g)
- Avocado (40 g)
- Olive oil (5 g)
- Pear (110 g)
- Hazelnut (20 g)

AM snack

- Banana (125 g)
- Walnuts (15 g)
- Raisins (15 g)

Lunch

- Steamed brassicas (kale 100 g, Brussels sprouts 100 g, broccoli 100 g, vinegar 15 g)
- Coalfish in green sauce (Coalfish 267 g, olive oil 10 g, mussels 100 g, garlic 4 g, parsley 1.5 g, white wine 26 g)
- Dried figs (75 g)
- Orange (180 g)

PM snack

- Apple (200 g)
- Dried plum (125 g)

Dinner

- Stewed beef (lean beef 167 g, olive oil 7 g, garlic 1.5 g, parsley 1.5 g, onion 42 g, black pepper 0.5 g, laurel 0.25 g, water 84 g)
- Steamed tubers (potato 100 g, sweet potato 100 g, carrot 50 g, chard 100 g)
- Pineapple (225 g)

**WEDNESDAY**

Breakfast

- Banana smoothie (banana 225 g and orange juice 200 ml)
- Grilled turkey breast with salad (turkey breast 90 g, tomato 90 g and avocado 40 g)

AM snack

- Strawberry (100 g)
- Dried fig (75 g)
- Walnut (15 g)

Lunch

- Grilled tubers (potato 125 g, sweet potato 100 g, carrot 75 g, pumpkin 75 g)
- Mero (Atlantic goliath grouper) a la bilbaína (Mero 175 g, garlic 3 g, bell pepper 10 g, olive oil 10 g and water 15 ml)
- Apple (250 g)

PM snack

- Almonds (25 g)
- Raisins (40 g)

Dinner

- Rabbit al estragon (rabbit 250 g, olive oil 3.5 g, white wine 20 g, tomato 63 g, garlic 1 g, onion 25 g, oregano 0.25 g and black pepper 0.5 g)
- Salad (lettuce 50 g, cucumber 50 g, tinned tuna 82 g, dates 50 g, white asparagus 75 g, avocado 40 g, basil 5 g, lemon juice 5 g and pickles 50 g)

**THURSDAY**

Breakfast

- Omelette (egg 55 g and olive oil 5 g)
- Tomato (100 g)
- Almonds (18 g)
- Banana (160 g)

AM snack

- Dried apricots (50 g)
- Pear (170 g)

Lunch

- Stewed beef (lean beef meat 150 g; potato 150 g; white wine 15 g; olive oil 10 g; onion 10 g; tomato 150 g and carrot 20 g)
- Steamed brassicas (kale 100 g, Brussels sprouts 100 g, broccoli 100 g, vinegar 15 g)
- Orange (180 g)

PM snack

- Banana and strawberry smoothie (banana 225 g; strawberry 100g, basil 5g and orange juice 200 ml)
- Dried plums (25 g)

Dinner

- Tuna in the oven (tuna 167 g; onion 25 g; olive oil 8.3 g; tomato-tinned and peeled- 160 g; lemon 33 g; red bell pepper 60 g; black pepper 1 g; garlic 2 g and white wine 15 g)
- Steamed tubers (potato 100 g; sweet potato 100 g; carrot 50 g; kale 50g and chard 100 g)

**FRIDAY**

Breakfast

- Peach (150 g)
- Apple (150 g)
- Raisins (25 g)
- Almonds (18 g)

AM snack

- Grilled turkey breast with salad (turkey breast 90 g; lettuce 50 g; tomato 100 g and avocado 40 g)
- Dried fig (25 g)

Lunch

- Garlic chicken with tomato* (chicken 175 g; onion 25 g; white wine 10 g; olive oil 15 g; garlic 7 g and tomato 150 g)
- Grilled tubers (potato 125 g, sweet potato 100 g, carrot 75 g, pumpkin 75 g and mushrooms 20 g)
- Orange (180 g)

PM snack

- Banana (160 g)
- Dates (25 g)
- Tangerine (60 g)
- Pear (110 g)

Dinner

- Baked salmon (salmon 150 g, honey 25g and dill 2 g)
- Sweet potato pure (sweet potato 250 g, onion 75 g, bell pepper 75 g, olive oil 5 g, water 15 ml, black pepper 2 g)

**SATURDAY**

Breakfast

- Avocado (40 g)
- Tomato (25 g) with oregano 1g and olive oil (5 ml)
- Melon (150 g)
- Dried figs (25 g)
- 1 boiled egg (55 g)
- Tinned tuna (56 g)

AM snack

- Dried plums (50 g)
- Pineapple (150 g)
- Banana (125 g)
- Hazelnuts (30 g)

Lunch

- Hake (Abadejo o merluza) with green sauce (hake 150 g; parsley 12 g; garlic 6 g; olive oil 15 g and water 62 g)
- “Campera” salad (potato 180 g; tomato 75 g; tinned tuna 20 g; green olives 19 g; onion 6 g; vinegar 5 g and black pepper 1 g)
- Apple (150 g)

PM snack

- Dried fig (25 g)
- Almonds (18 g)
- Pear (110 g)
- Pineapple (150 g)

Dinner

- Boiled angler with orange (angler 150 g; white wine 20 g; black pepper 0.5 g; laurel 0.25 g; onion 25 g; orange 56 g and dill 0.25 g)
- Grilled tubers (potato 125 g, sweet potato 100 g, carrot 75 g, pumpkin 75 g)
- Dried plum (25 g)
- Dried apricot (25 g)
- Dates (25 g)

**SUNDAY**

Breakfast

- Banana smoothie (banana 225 g; basil 5 g and orange juice 200 ml)
- Grilled turkey breast with salad (turkey breast 90 g, tomato 90 g and avocado 40 g)

AM snack

- Omelette with tomato and avocado (egg 55 g; tomato 100 g and avocado 40 g)
- Tangerine (85 g)

Lunch

- Baked coalfish (coalfish 150 g; prawns 40 g; green beans 28 g; leek 20 g; squash 25 g; potato 150 g; carrot 100 g; onion 68 g; white wine 25 g; olive oil 7 g and small onion 19 g)
- Steamed brassicas (kale 100 g, Brussels sprouts 100 g, broccoli 100 g, vinegar 15 g)
- Banana (225 g)

PM snack

- Banana 225 g
- Orange juice 200 g

Dinner

- Grilled chicken breast (chicken breast 75 g and olive oil 5 g)
- Salad (lettuce 50 g, cucumber 50 g, tinned tuna 56 g, dates 50 g, white asparagus 75 g, avocado 40 g; tomato 75 g and pickles 50 g)
- Dried fig (25 g)
- Dried plum (25 g)
